# Neural markers of errors as endophenotypes in neuropsychiatric disorders

**DOI:** 10.3389/fnhum.2013.00350

**Published:** 2013-07-18

**Authors:** Dara S. Manoach, Yigal Agam

**Affiliations:** ^1^Department of Psychiatry, Massachusetts General Hospital and Harvard Medical SchoolBoston, MA, USA; ^2^Athinoula A. Martinos Center for Biomedical ImagingCharlestown, MA, USA

**Keywords:** error-related negativity, anterior cingulate, error processing, response monitoring, imaging genetics

## Abstract

Learning from errors is fundamental to adaptive human behavior. It requires detecting errors, evaluating what went wrong, and adjusting behavior accordingly. These dynamic adjustments are at the heart of behavioral flexibility and accumulating evidence suggests that deficient error processing contributes to maladaptively rigid and repetitive behavior in a range of neuropsychiatric disorders. Neuroimaging and electrophysiological studies reveal highly reliable neural markers of error processing. In this review, we evaluate the evidence that abnormalities in these neural markers can serve as sensitive endophenotypes of neuropsychiatric disorders. We describe the behavioral and neural hallmarks of error processing, their mediation by common genetic polymorphisms, and impairments in schizophrenia, obsessive-compulsive disorder, and autism spectrum disorders. We conclude that neural markers of errors meet several important criteria as endophenotypes including heritability, established neuroanatomical and neurochemical substrates, association with neuropsychiatric disorders, presence in syndromally-unaffected family members, and evidence of genetic mediation. Understanding the mechanisms of error processing deficits in neuropsychiatric disorders may provide novel neural and behavioral targets for treatment and sensitive surrogate markers of treatment response. Treating error processing deficits may improve functional outcome since error signals provide crucial information for flexible adaptation to changing environments. Given the dearth of effective interventions for cognitive deficits in neuropsychiatric disorders, this represents a potentially promising approach.

To adapt to the environment, human beings must learn from the consequences of their behavior. Understanding the nature of the brain mechanisms that flexibly modify behavior based on its consequences is a fundamental goal of neuroscience. These mechanisms are also of considerable clinical importance since a number of neuropsychiatric disorders are strongly associated with maladaptively rigid and repetitive behaviors that are not optimally responsive to outcomes. One approach to understanding the neural basis of learning from consequences is to study error processing. Errors provide critical information for adjusting behavior to optimize outcomes. Error processing, which is also referred to as “response monitoring” or “performance monitoring,” involves detecting errors during task performance, evaluating what went wrong, and adjusting behavior accordingly. These dynamic adjustments of responses are at the heart of behavioral flexibility. They enable individuals to optimize function in complex, uncertain, and constantly changing environments. Since learning from errors is impaired in several neuropsychiatric disorders, understanding the neural and genetic mechanisms of error processing has important clinical implications. Identifying specific deficits can illuminate the pathophysiology of these disorders and provide novel targets for treatment. Below, we selectively review the behavioral and neural hallmarks of error processing; impairments in schizophrenia, obsessive-compulsive disorder, and autism spectrum disorders (ASDs); and genetic contributions. The goal is to evaluate the potential of the neural markers of errors to serve as endophenotypes. Endophenotypes are biologically-based heritable dysfunctions that are thought to be a closer reflection of the effects of the genes that predispose to illness than either the diagnosis itself, or the symptoms that define it (Gottesman and Gould, 2003). The identification of clinically-relevant endophenotypes can facilitate the discovery of susceptibility genes, mechanisms of illness, and targets for intervention (Hariri et al., 2006).

## Behavioral indices of error processing

Both the behavioral and neural markers of error processing are considered to be “generic” in that they are elicited by a wide range of tasks regardless of response modality (Holroyd and Coles, 2002). Many experimental tasks used to study error processing in humans require response inhibition, or the suppression of prepotent but contextually inappropriate responses. These include variations of go no-go, antisaccade (Hallett, 1978), countermanding or stop-signal (Logan and Cowan, 1984), Stroop (1935), Simon (1969), and perhaps most commonly, Eriksen flanker (Eriksen and Eriksen, 1974) tasks.

**Figure 1:**
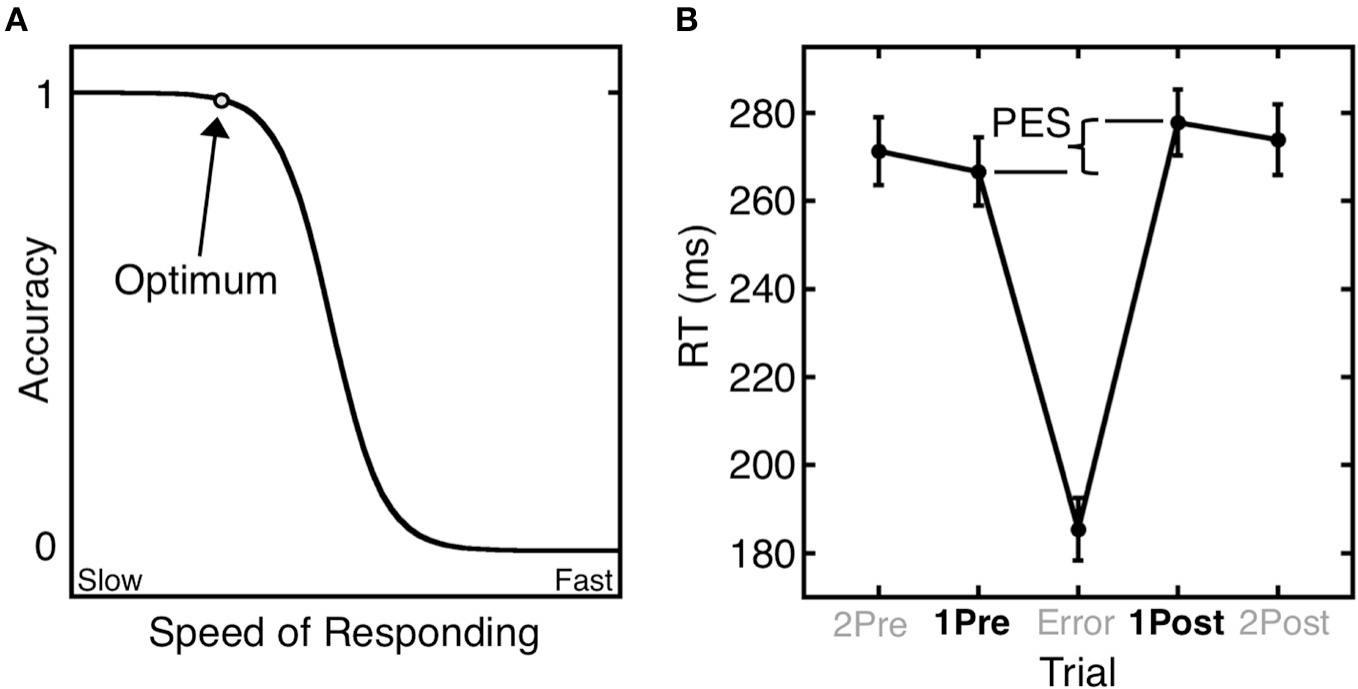
**Trial-by-trial adjustments of reaction time (RT). (A)** A schematic depiction of the SATO function. The circle denotes the optimum: the point at which the highest accuracy is achieved at the fastest possible speed. Beyond this point, speedier responses entail a cost (trade-off) in reduced accuracy. **(B)** Mean saccadic RT during an antisaccade task as a function of trial position relative to an error trial. Post-error slowing (PES) is defined as the difference in RT between the trial following the error (1Post) and the trial preceding the error (1Pre). Error bars represent the standard error of the mean.

Errors give rise to both immediate and longer-term remedial adjustments of behavior. Short-term, or trial-by-trial adjustments include the immediate self-correction of errors and the slowing of reaction time (RT) in trials that follow an error (i.e., post-error slowing) (Rabbitt, 1966). These trial-by-trail adjustments of RT based on error history are well-described by the Speed-Accuracy Trade-Off (SATO) function. The SATO function depicts the non-linear relation between speed and accuracy such that faster responding does not affect accuracy, but only up to a point. Beyond that point, speed and accuracy are inversely related, with slower responses having a greater probability of being correct (Figure 1A). This transition point can be regarded as an optimum, where the best accuracy is achieved at the fastest possible speed. Over trials, responses speed up until an error is committed (Ridderinkhof et al., 2003), and following an error, RT slows, and the probability of an error decreases (Figure 1B). This pattern can be interpreted as a progression to riskier positions on the SATO function culminating in an error. The error is followed by a shift back to a safer position on the function that has a greater likelihood of a correct response.

Reinforcement learning theory (Thorndike, 1911) can be invoked to account for longer-term behavioral changes in response to errors. Its main principle is that rewarded actions are more likely to be repeated, while actions with negative consequences are less likely to recur. In behavioral terms, reinforcement learning involves the strengthening or weakening of stimulus-response mappings based on behavioral outcomes. While reinforcement learning has traditionally been studied using explicit rewards and punishments, more recent theory extends it to errors (Holroyd and Coles, 2002). Errors on cognitive tasks are both salient (in that they are often unexpected) and aversive (representing the non-achievement of a goal). As failures of performance they often have negative consequences. For these reasons, errors prompt reinforcement learning.

## Neural markers of errors, their functional significance and relations to one another

Electrophysiological and neuroimaging studies have identified two highly reliable neural markers of error commission—the error-related negativity (ERN) and functional MRI (fMRI) activation of the dorsal anterior cingulate cortex (dACC; Taylor et al., 2007)—that are the focus of the present review. Although these error markers have been extensively studied, their functional significance and relations to one another are incompletely understood.

### The error-related negativity (ERN)

**Figure 2:**
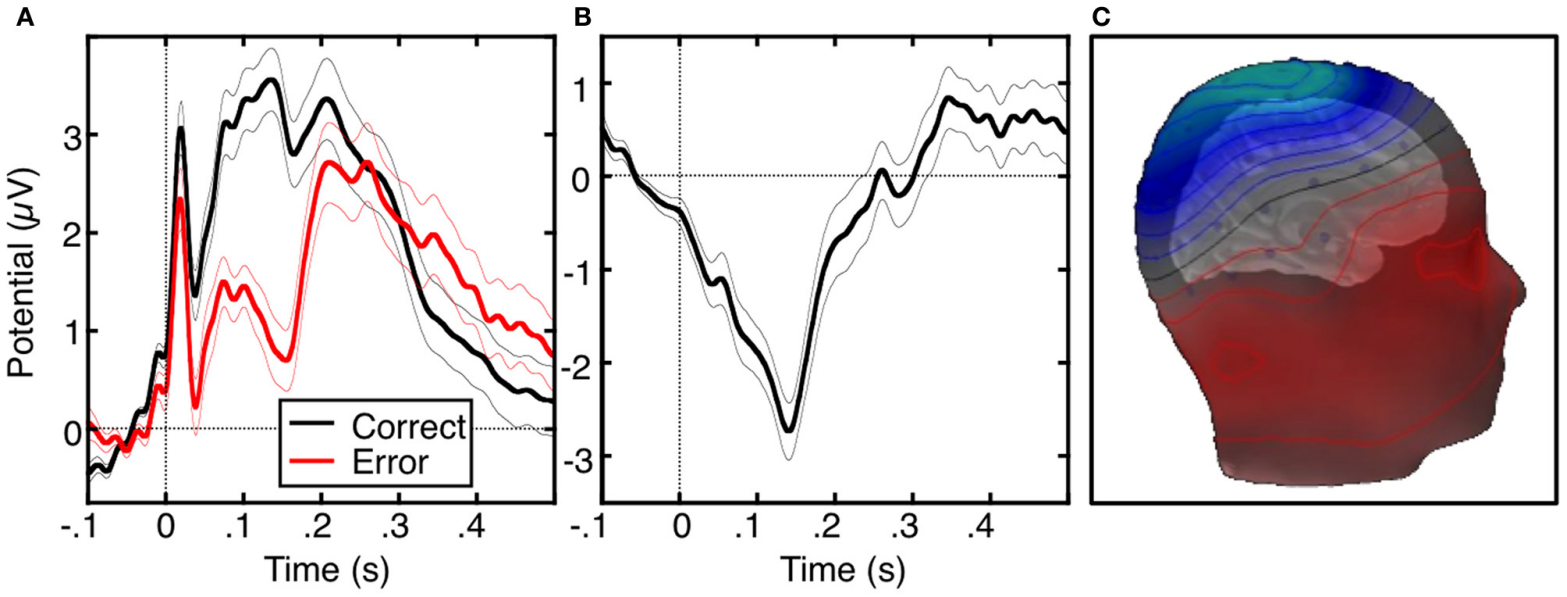
**The error-related negativity (ERN). (A)** Grand average waveforms for correct (black) and error (red) antisaccade trials, time-locked to the onset of the saccade. **(B)** Difference waveform, obtained by subtracting the correct waveform from the error waveform. **(C)** Scalp distribution of the ERN, displayed on a template head model. Adapted from Agam et al. (2011).

The ERN or error negativity (Ne) is an event-related potential that peaks ~100 ms following an error (Figure 2, Falkenstein et al., 1991; Gehring et al., 1993; Dehaene et al., 1994; Van Veen and Carter, 2002) and is usually measured on the scalp with electroencephalograpy (EEG), magnetoencephalography (MEG; Keil et al., 2010) or a combination of both techniques (Agam et al., 2011). The ERN is usually defined as the peak of the difference between the averaged waveforms of error and correct trials time-locked to the onset of the response. The ERN is the earliest error marker and is “generic” in that it is seen across a variety of behavioral paradigms and response modalities. Comparisons of ERNs time-locked to button presses, saccadic eye movements, or foot presses, reveal a similar morphology, amplitude and scalp topography (Holroyd et al., 1998; Van 'T Ent and Apkarian, 1999). ERN latency, however, varies based on the measurement technique. Button presses elicit shorter latencies than ERNs locked to the electromyography (EMG) or saccadic responses as measured by electrooculography (EOG). This reflects that EMG and EOG measure the onset of movement, which occurs earlier than its outcome (e.g., a button press). The ERN is usually maximal at electrode Cz on the scalp (e.g., Van Veen and Carter, 2002; Van Schie et al., 2004; Agam et al., 2011), but the peak location can be more anterior (e.g., Gehring and Fencsik, 2001; Nieuwenhuis et al., 2003; Endrass et al., 2005) or posterior (e.g., Hajcak et al., 2004 Van Boxtel et al., 2005; Ladouceur et al., 2007) and factors such as response modality and task fail to provide a convincing account of this variability.

The ERN has been proposed to reflect error detection and reinforcement learning (Paus et al., 1993; Holroyd and Coles, 2002; Holroyd et al., 2004b). Its amplitude is greater when accuracy is emphasized over speed (Gehring et al., 1993), when errors are corrected (Scheffers and Coles, 2000), when errors incur greater loss (Holroyd et al., 2004a) and when errors are less frequent and therefore also less expected (Gehring et al., 1993; Hajcak et al., 2003). Larger ERNs are associated with greater post-error slowing of responses (Debener et al., 2005) and ERN latency predicts the speed of self-corrections (Fiehler et al., 2005). These findings suggest that the ERN indicates error detection, is sensitive to both the predictability and value of outcomes, and contributes to dynamic, trial-by-trial adjustments of performance.

### Error positivity (Pe)

A second EEG error marker warrants consideration given its relevance to neuropsychiatric disorders. The error positivity or Pe (Van Veen and Carter, 2002) is an event-related potential that occurs ~300–500 ms following an error (for review see, Overbeek et al., 2005). The Pe has been localized to the rostral anterior cingulate cortex (Van Veen and Carter, 2002; Van Boxtel et al., 2005), though one study reported a dACC source (Herrmann et al., 2004). The Pe is not as well characterized and is less consistently observed than the ERN, which may reflect that it is a later and more variable component of error processing. While the ERN is present regardless of whether an error was perceived, the Pe is present only for perceived errors and is thought to index error awareness (Nieuwenhuis et al., 2001; Endrass et al., 2007). The Pe has also been associated with the subjective or emotional appraisal of errors (Van Veen and Carter, 2002) and with short-term performance adjustments such as error correction and post-error slowing (Nieuwenhuis et al., 2001).

### Error-related FMRI activation of the anterior cingulate cortex (ACC)

**Figure 3:**
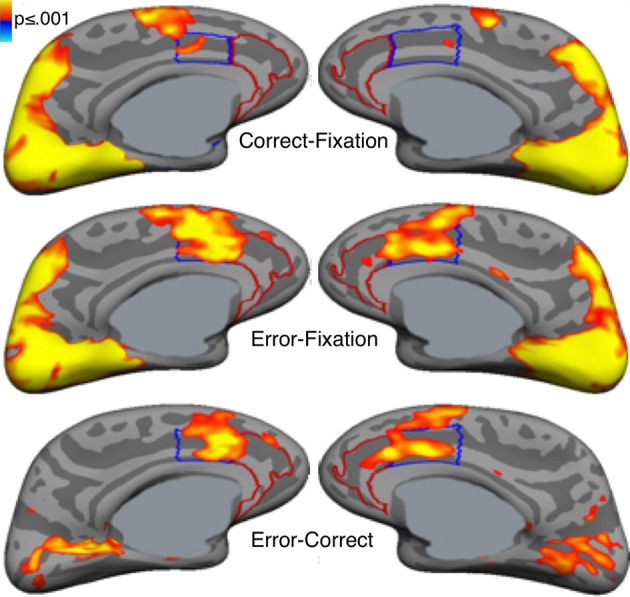
**Error-related activation in the anterior cingulate cortex (ACC).** Statistical maps, displayed on medial cortical surface templates, show activation on correct trials vs. a fixation baseline (top), error vs. fixation (middle) and error vs. correct (bottom). Gray masks cover subcortical regions in which activation is displaced in a surface rendering. The dACC and rACC are outlined in blue and red, respectively. Adapted from Polli et al. (2005).

Error commission is also reliably associated with increased fMRI activation of the ACC on error compared with correct trials (i.e., error-related activation, Figure 3; for review see, Taylor et al., 2007). The ACC can be divided into a dorsal region (dACC) that extends caudally from the genu of the corpus callosum to the vertical plane of the anterior commissure, and interacts with the striatum and other cortical regions to mediate motor and cognitive processing, and a rostral region (rACC) that lies anterior and ventral to the genu of the corpus callosum and interacts with other paralimbic and limbic regions, including the amygdala and insula, to mediate emotional processing (Devinsky et al., 1995; Bush et al., 1998, 2000; Whalen et al., 1998; Phillips et al., 2003). Like the Pe, error-related rACC activation is thought to reflect appraisal of the affective or motivational significance of errors (Van Veen and Carter, 2002; Luu et al., 2003; Taylor et al., 2006). Such appraisal may also involve the insula and amygdala, both of which are densely interconnected with the rACC (Van Hoesen et al., 1993) and show increased activity with errors (Menon et al., 2001; Brazdil et al., 2002; Garavan et al., 2002; Polli et al., 2009). While both dACC and rACC show error-related activation (Van Veen and Carter, 2002; Luu et al., 2003; Taylor et al., 2006), dACC activation is more consistently observed. Like the ERN, greater error-related dACC activation is associated with lower error rates (Polli et al., 2008; Fitzgerald et al., 2010) and increased post-error slowing (Garavan et al., 2002; Kerns et al., 2004; Klein et al., 2007a).

### Modulation of default network activation in relation to errors

The brain's default network is thought to mediate self-referential and affective processing and is usually deactivated during effortful cognitive tasks (Raichle et al., 2001; Buckner et al., 2008). During error trials (Polli et al., 2005) and trials immediately preceding errors (Li et al., 2007; Eichele et al., 2008), however, the default network shows relatively increased activation, which may reflect increased focus on the internal milieu at the expense of attention to the task (Drevets and Raichle, 1998). In trials that follow errors, task-induced deactivation is re-established (Eichele et al., 2008). This cyclical pattern of default network activation in trials including and surrounding errors correlates with SATO based changes in RT (i.e., pre-error speeding, faster errors, and post-error slowing, Agam et al., 2013) and suggests that interference from internally-directed thought culminates in an error, which, in turn prompts renewed attention to the task in the subsequent trial. These changes in activation are not strictly error markers (i.e., they are not specific to errors nor do they necessarily indicate that an error has occurred), but they may contribute to error commission and to behavioral adjustments following errors such as post-error slowing. Several reviews have addressed the role of default network function in neuropsychiatric disorders (e.g., Buckner et al., 2008; Broyd et al., 2009; Sandrone, 2012; Whitfield-Gabrieli and Ford, 2012). Whether changes of default network activity in relation to errors are affected in neuropsychiatric disorders, however, is largely unexplored.

### Error-based reinforcement learning

Error-related dACC activation is often assumed to be the hemodynamic correlate of the ERN. This assumption is consistent with both EEG and MEG studies that have reported a dACC source for the ERN and with models that attribute both error markers to a specific neural mechanism that implements error-based reinforcement learning (Holroyd and Coles, 2002; Ridderinkhof et al., 2004; Taylor et al., 2007). Consistent with animal neurophysiology and human neuroimaging findings, these models view the neural sequelae of error commission as indices of error-based reinforcement learning (Holroyd and Coles, 2002; Schultz, 2002). When an error occurs, the striatum detects a mismatch between the intended (correct) versus actual (error) outcome. This mismatch or “prediction error” results in a phasic decrease in mesencephalic dopamine (DA) release that results in the disinhibition of neurons in the dACC. These neurons generate the ERN. According to this theory, both increased dACC activation and the ERN reflect the use of DA-dependent error signals to modify the associative strength of stimulus-response mappings in the service of optimizing behavioral outcomes (Holroyd et al., 2003, 2004b). Thus, both error-related dACC activation and ERN can be conceptualized as DA-dependent training signals that are used to learn from errors (Holroyd and Coles, 2002; Brown and Braver, 2005). Similar neural mechanisms of error processing have been observed across species for a variety of learning tasks. For example, the songbird uses input from a basal ganglia—thalamocortical circuit to recognize and correct vocal errors while learning its distinctive song (Andalman and Fee, 2009). Such findings suggest that this neural circuitry represents an evolutionarily conserved mechanism for learning from errors.

### Relation of the ERN to dACC activation

Despite the many studies that report a dACC source for the ERN, the location of the neural generator of the ERN is still a topic of debate. When compared across studies, the dACC source loci of the ERN show considerable variation (for review, see Agam et al., 2011) and all are posterior to the mean location of error-related fMRI activation (based on a meta-analysis of 13 fMRI studies, Ridderinkhof et al., 2004). Some ERN loci also fall in the posterior cingulate cortex (PCC) according to standard anatomical definitions that place the ACC/PCC border between *y* = −2 and *y* = −12 mm in Talairach space (Bush et al., 2000). The PCC is also a plausible generator of the ERN. It shows error-related fMRI activation (Menon et al., 2001; Fassbender et al., 2004; Wittfoth et al., 2008), though not nearly as consistently as the dACC, and like the ERN, its activity is modulated by the value of behavioral outcomes (McCoy et al., 2003; Fujiwara et al., 2009; Smith et al., 2009). An MEG study reported a PCC source for the feedback-related negativity, which is thought to be generated by the same generic mechanism as the ERN (Donamayor et al., 2011). Further, a study from Agam and colleagues that combined data from EEG and MEG, localized the source of the ERN to the PCC (Agam et al., 2011). This PCC region was clearly distinct from error-related dACC activation measured in the same participants performing the same task during fMRI.

These findings challenge the view that dACC activation and the ERN are different measurements of the same underlying neural mechanism. Instead, they indicate that the ERN and fMRI activation of the dACC reflect distinct neural responses to errors. In the combined MEG/EEG, fMRI, and diffusion tensor imaging (DTI) study of Agam and colleagues, ERN amplitude correlated with fMRI activation in both the PCC and dACC, and these two regions showed coordinated activity based on functional connectivity MRI. This suggests that the dACC and PCC are components of a functional network that mediates error processing. The PCC and ACC have direct anatomical connections through the cingulum bundle (Schmahmann et al., 2007) and increased microstructural integrity of the posterior cingulum bundle (as indexed by DTI measurements of fractional anisotropy) predicted faster error self-correction. To the degree that fractional anisotropy reflects myelination, increased myelination along the cingulum bundle may speed the conduction of the message that an error has occurred thereby resulting in faster corrective responses. Taken together, these findings are consistent with the theory that the PCC detects errors, gives rise to the ERN, and then relays error information to the dACC via the cingulum bundle to implement corrective behavior. Refinements of this working model will likely follow given that the mechanisms of error processing remain a highly active area of research.

## Error processing impairments in neuropsychiatric disorders

Although the present review focuses on schizophrenia, obsessive-compulsive disorder (OCD) and ASD, accumulating evidence suggests that error processing deficits contribute to rigid, repetitive behavior in a range of disorders. For example, a previous review described ERN abnormalities in anxiety disorders, depression and substance abuse and their relations to symptoms (Olvet and Hajcak, 2008). Emerging evidence also indicates that error processing deficits differ by diagnosis suggesting distinct neural mechanisms and genetic contributions. This has important implications for understanding pathophysiology and for the treatment of associated cognitive and behavioral dysfunction. Below, we evaluate evidence that neuroimaging-based markers of deficient error processing can serve as sensitive endophenotypes of neuropsychiatric disorders.

### Schizophrenia

Perseveration, or the contextually inappropriate and unintentional repetition of responses, is a classic behavioral abnormality in schizophrenia. At least some forms of perseveration may reflect a failure to use error feedback to guide behavior. A classic example is continuing to make a previously reinforced response on the Wisconsin Card Sort Test even though feedback indicates that it is no longer correct (e.g., Goldberg et al., 1987). These perseverative errors reflect both motivational and cognitive factors (Summerfelt et al., 1991) and exemplify the behavioral rigidity despite changing contingencies that is often observed in schizophrenia.

Both neuroimaging and electrophysiological studies consistently report blunted neural responses to errors in schizophrenia. fMRI studies show reduced error-related dACC and rACC activation (Carter et al., 2001; Laurens et al., 2003; Kerns et al., 2005). Reduced error-related activation extends to “reinforcement learning circuitry,” comprising the dACC, substantia nigra, caudate, and putamen, and to “affective appraisal circuitry” comprising the rACC, insula, and amygdala, in which reduced activation may reflect diminished concern regarding behavioral outcomes (Polli et al., 2008). These reductions remain after statistically controlling for the effects of antipsychotic medication dose and error rate, the latter indicating that the blunted neural response to errors in schizophrenia is not simply a reflection of more frequent, and therefore more predictable errors.

Patients with schizophrenia also consistently show a blunted ERN (Kopp and Rist, 1999; Alain et al., 2002; Bates et al., 2002; Mathalon et al., 2002; Morris et al., 2006; Foti et al., 2012; Perez et al., 2012). Even in the context of an abnormal ERN, however, the Pe is intact in patients in many (Alain et al., 2002; Mathalon et al., 2002; Morris et al., 2006; Simmonite et al., 2012) but not all studies (Foti et al., 2012; Perez et al., 2012). Immediate error-related performance adjustments such as post-error slowing and error self-correction are also often intact (Kopp and Rist, 1994, 1999; Levy et al., 1998; Mathalon et al., 2002; Laurens et al., 2003; Polli et al., 2006, 2008), although impaired performance adjustments have also been reported (Malenka et al., 1982, 1986; Carter et al., 2001; Turken et al., 2003). Dissociations between intact performance adjustments and reduced ACC activity and ERN amplitude are often seen within single studies (Kopp and Rist, 1999; Mathalon et al., 2002; Laurens et al., 2003; Polli et al., 2008) and suggest that error processing deficits in schizophrenia are selective.

Findings of blunted ERN and dACC activation in schizophrenia are remarkably consistent and may reflect a more general problem with reinforcement learning, which is impaired in schizophrenia (Waltz and Gold, 2007; Waltz et al., 2007, 2010). They may also reflect functional and structural abnormalities of the cingulate cortex. There is overwhelming evidence of abnormal ACC function and structure in schizophrenia including gray matter abnormalities (e.g., Ohnuma et al., 1997; Goldstein et al., 1999; Sigmundsson et al., 2001; Suzuki et al., 2002; Kuperberg et al., 2003; Ha et al., 2004; Yamasue et al., 2004; Mitelman et al., 2005), volume reductions in the white matter underlying the ACC (McDonald et al., 2005; Mitelman et al., 2005) and reduced fractional anisotropy of white matter underlying the cingulate cortex in many (Ardekani et al., 2003; Kubicki et al., 2003; Sun et al., 2003; Wang et al., 2004; Hao et al., 2006; Manoach et al., 2007) but not all studies (Buchsbaum et al., 1998; Agartz et al., 2001; Foong et al., 2002; Burns et al., 2003). Histopathological studies give evidence of disturbances in ACC micro- and macro-circuitry that might alter communication with connected regions (e.g., Benes, 1993, 2000), consistent with reports of reduced functional and structural connectivity of the ACC in schizophrenia (e.g., Manoach et al., 2007; Tu et al., 2010; Kyriakopoulos et al., 2012; Yan et al., 2012).

Treatment with antipsychotic drugs is an important confound in this literature given its effects on dopamine neurotransmission and indices of error processing (e.g., Zirnheld et al., 2004). Several lines of evidence suggest that deficient error processing is not merely a side-effect of treatment. Functional and structural ACC abnormalities, which predict the onset of psychosis (Fornito et al., 2008), are seen in never-medicated high-risk youth (Whalley et al., 2006), and in never-medicated children experiencing psychotic symptoms (Jacobson et al., 2010). In addition, a blunted ERN, similar to that observed in schizophrenia, is seen in syndromally-unaffected siblings (Simmonite et al., 2012), in never-medicated children with putative antecedents to schizophrenia (Laurens et al., 2010) and in antipsychotic naïve patients at high clinical risk for psychosis (Perez et al., 2012). These studies suggest that antipsychotic drugs do not fully account for blunted error processing or functional and structural ACC abnormalities in schizophrenia. Instead, this literature suggests that ACC abnormalities and error processing deficits are trait markers of genetic vulnerability to schizophrenia that predate the onset of illness. Impairments in evaluating and learning from errors in schizophrenia may substantially contribute to the rigid, perseverative, and maladaptive patterns of thought and behavior that characterize schizophrenia and compromise social and occupational function (Kim et al., 2006). In support of this possibility, a recent study reported that a blunted ERN was associated with more severe negative symptoms and poorer real world function as indicated by unemployment and re-hospitalization (Foti et al., 2012).

### Obsessive-compulsive disorder (OCD)

OCD is characterized by uncontrollable, unwanted thoughts (i.e., obsessions) and repetitive, ritualized behaviors that individuals feel compelled to perform (compulsions). In contrast to the blunted neural responses to errors in schizophrenia, OCD is often associated with exaggerated error responses including increased error-related ACC activation (Ursu et al., 2003; Fitzgerald et al., 2005, 2010; Maltby et al., 2005) and increased ERN amplitude not only on error trials (Gehring et al., 2000; Johannes et al., 2001; Ruchsow et al., 2005; Santesso et al., 2006; Endrass et al., 2008, 2010; Xiao et al., 2011) but also on correct trials in some (Ursu et al., 2003; Maltby et al., 2005), but not all studies (Gehring et al., 2000; Fitzgerald et al., 2005). One study reported a normal ERN to errors in OCD (e.g., Nieuwenhuis et al., 2005) and recent findings (Kaczkurkin, 2013) including those of a meta-analysis (Mathews et al., 2012) suggest that while the ERN is generally increased, this varies based on the type of task, the level of difficulty and the symptoms present. A recent study of children with OCD found an increased ERN in both patients and their unaffected siblings relative to controls suggesting that the ERN is a marker of genetic risk for OCD (Carrasco et al., 2013). Both increased ERN amplitude (Gehring et al., 2000) and error-related ACC activation (Ursu et al., 2003; Fitzgerald et al., 2005) have been associated with the severity of obsessions and compulsions in OCD suggesting that hyperactive error processing contributes to its defining features. This hypothesis is consistent with a longstanding theory of OCD that inappropriate and exaggerated error signals in response to behavioral outcomes lead to a pervasive sense of incompleteness and self-doubt (Pitman, 1987) that triggers the compulsion to repeat behaviors, even if they were already successfully completed (Maltby et al., 2005). In this scenario, an individual suffering from OCD may remember correctly that they locked the door, but inappropriate and persistent error signals may indicate that something is “not quite right” and compel them to check repeatedly that the door is indeed locked. Findings that the ACC and connected regions show increased activation during symptom provocation in OCD (Breiter et al., 1996), and that cingulotomy relieves obsessions and compulsions (Dougherty et al., 2002) also support the link between hyperactivity in ACC circuitry and rigid, repetitive behaviors.

Measurements of obsessive-compulsive behavior have also been related to indices of error processing in non-clinical samples. Obsessive characteristics are related to the amplitudes of the ERN and Pe in children (Santesso et al., 2006) and to the amplitude of the ERN in college undergraduates (Hajcak and Simons, 2002), suggesting that obsessive-compulsive traits in the general population are mediated by error processing mechanisms.

### Autism spectrum disorders (ASDs)

ASDs are neurodevelopmental disorders characterized by three core features: impaired social interaction, impaired communication, and restricted, repetitive and stereotyped patterns of behavior, interests and activities. Although repetitive and restricted behaviors are often the most disabling feature of ASD (Bishop et al., 2007) they have received the least research attention. They are present as early as 18 months, predict outcome independently of social and communication deficits, and may interfere with the development of social and communication skills that are deficient in ASD (Morgan et al., 2008; Watt et al., 2008). The hypothesis that error processing deficits characterize ASD and contribute to behavioral repetition and rigidity receives only mixed support from the literature.

Several studies have reported a blunted ERN in ASD (Vlamings et al., 2008; Sokhadze et al., 2010, 2012b; South et al., 2010; Santesso et al., 2011), one has reported normal ERN (Groen et al., 2008), and yet another found an increased latency (and amplitude in a high functioning subset of participants) of the ERN (Henderson et al., 2006). The finding that repetitive low frequency transcranial magnetic stimulation (rTMS) to bilateral dorsolateral prefrontal cortex in high functioning children with ASD was associated with an increased ERN (but also a decreased error rate), suggests the possibility of intervention to modulate error processing (Sokhadze et al., 2012a).

Behaviorally, reduced error self-correction (Russell and Jarrold, 1998), normal rates of error self-correction (Thakkar et al., 2008), and reduced post-error slowing (Bogte et al., 2007) have all been observed. Two fMRI studies reported exaggerated error-related ACC activation in ASD (Thakkar et al., 2008; Goldberg et al., 2011) and in one of these, increased ACC activation on correct trials that correlated with higher clinical ratings of restricted, repetitive behavior in ASD, thus linking abnormal error processing to a core symptom (Thakkar et al., 2008). This relation may reflect that reduced discrimination between correct and error outcomes interferes with adjusting behavior to obtain the most favorable outcome. Another compatible possibility is that like OCD, in ASD uncomfortable error signals following correct responses compel repetitive behavior. In ASD these abnormal signals on correct trials were maximal in the rACC, which is thought to contribute to an appraisal of the affective or motivational salience of errors (Van Veen and Carter, 2002; Luu et al., 2003; Taylor et al., 2006). Finally, three studies, including the one reporting increased ACC activation on both error and correct trials, have reported reduced fractional anisotropy (FA) in ACC white matter as measured by DTI (Barnea-Goraly et al., 2004; Thakkar et al., 2008; Noriuchi et al., 2010), but not a fourth, which reported increased FA in ACC white matter (Cheng et al., 2010).

In summary, the literature provides only preliminary support for the hypothesis that cingulate cortex abnormalities impair error processing in ASD and contribute to restricted, repetitive behavior. At present, repetitive behaviors in ASD are incompletely understood and neurobiologically-valid dimensions have not been delineated. Efforts to understand the contribution of error processing to specific dimensions of repetitive behavior and to identify the underlying mechanisms can guide the development of targeted treatments.

## Rationale for the use of neuroimaging-based cognitive endophenotypes

**Figure 4:**
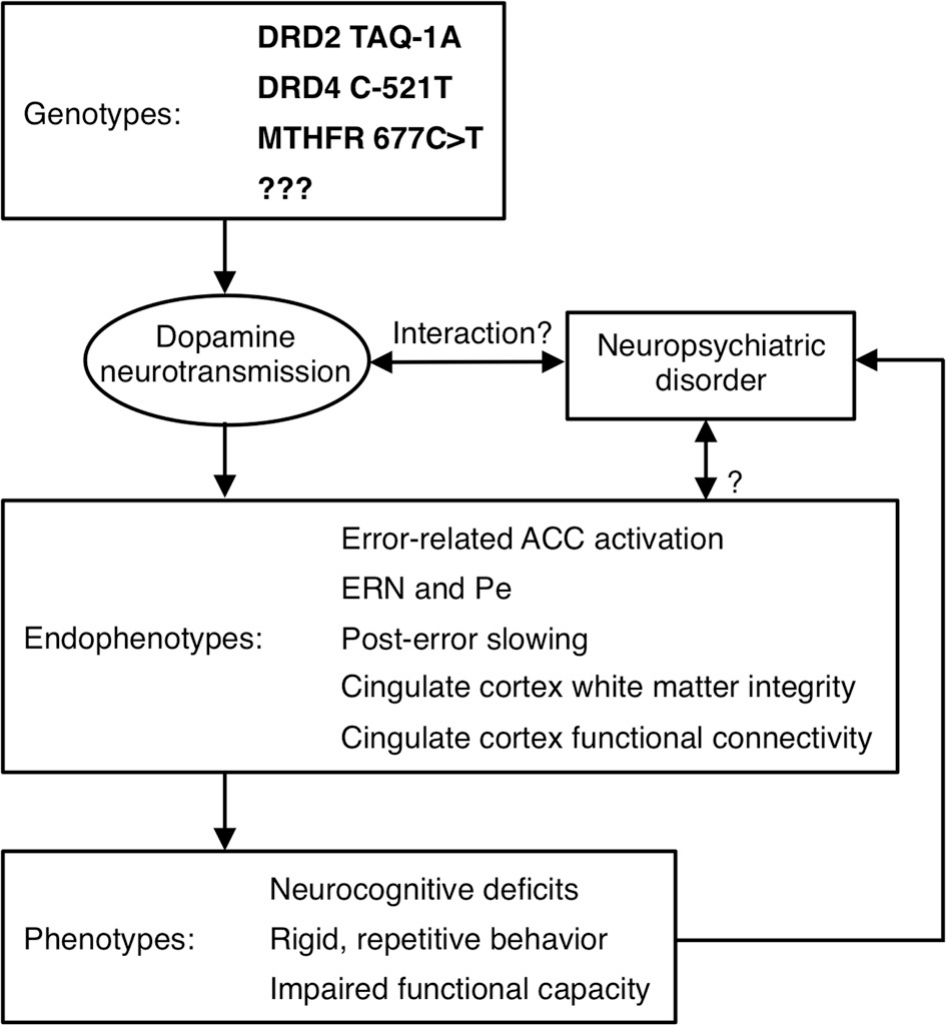
**Model of a causal pathway for error processing.** Specific genetic polymorphisms affect dopamine neurotransmission, which may interact with a neuropsychiatric disorder to affect neuroimaging-based endophenotypes. These endophenotypes, in turn, contribute to the expression of phenotypes, which may influence whether a psychiatric diagnosis is given.

Although Diagnostic and Statistical Manual (DSM) Axis I psychiatric disorders are highly heritable, their genetic origins remain elusive. A major obstacle to identifying genetic risk factors is the difficulty defining neurobiologically-valid phenotypes for inclusion in studies. Current DSM criteria for disorders such as schizophrenia and autism define phenotypes that are so broad that it is possible for two study samples with the same diagnosis to bear little resemblance to one another. This phenotypic heterogeneity suggests etiological and genetic heterogeneity and reliance on such overly broad diagnostic categories can lead to inconsistent findings. Within studies, relatively large effects may be obscured because they only characterize a subset of the sample. While phenotypic heterogeneity is expected within complex genetic disorders such as schizophrenia and autism, subdivision based on phenotypes has not led to neurobiologically-valid subtyping schemes. In schizophrenia, for example, most subtyping schemes have been based on symptoms (e.g., positive vs. negative, deficit vs. non-deficit, paranoid vs. non-paranoid), but symptom definitions are broad and imprecise and their assessment is heavily dependent on self-report. In addition, symptoms often lack temporal stability and predictive validity (i.e., they do not provide an adequate account of variability in other important measures such as brain structure or function, disease course, or functional outcome). Moreover, neither diagnosis nor symptoms can identify syndromally-unaffected relatives who carry susceptibility genes. Finally, the substantial shared genetic liability for neuropsychiatric disorders such as schizophrenia, major depressive disorder, ASD, attention deficit-hyperactivity disorder and bipolar disorder (e.g., Craddock et al., 2006a; Crespi et al., 2010; Purcell et al., 2009; Cross-Disorder Group of the Psychiatric Genomics et al., 2013), reinforces that our present diagnostic categories and symptom definitions do not map onto distinct underlying genetic etiologies. To the extent that genes *cause* psychiatric disorders and their signs and symptoms, they do so via their effects on brain function (Tan et al., 2008). Given the heterogeneity of present diagnostic categories, alternate phenotyping strategies are needed to understand the genetic origins and mechanisms of psychiatric disorders and to facilitate the development of more valid psychiatric nosology and more effective interventions. This imperative spurred the National Institute of Mental Health (NIMH) to implement a Research Domain Criteria Project, or “RDoC” (see http://www.nimh.nih.gov/research-funding/rdoc/index.shtml) strategy. The RDoC strategy involves developing, “…for research purposes, new ways of classifying mental disorders based on dimensions of observable behavior and neurobiological measures.” RDoC encourages researchers to base their selection of subjects on dimensions that can be characterized along the causal chain from genes to molecules to circuits to behavior, rather than relying on DSM diagnoses (Figure 4 illustrates a theoretical causal chain for error processing). “Cognitive Systems” is one of the broad domains identified by RDoC for study, and below we argue that neuroimaging-based measures of cognition are more sensitive indices of genetic mechanisms than behavior.

While is well-accepted that genetic variation influences brain function and contributes to cognitive deficits in neuropsychiatric disorders, genetically mediated alterations in brain function are not always manifest at the level of behavior. Preserved behavior may reflect the use of an alternate strategy and/or the recruitment of compensatory neural circuitry. Conversely, disordered behavior may reflect not only the brain function of interest, but deficits of other systems, including of the motor output systems that are required to produce the behavior. Thus, behavior is an indirect and possibly unreliable index of genetic effects on brain function. Because brain function is a more direct index of genetic mechanisms than behavior, neuroimaging-based endophenotypes can result in increased effect sizes in studies of genetic variation. Gene effects on functional and structural neuroimaging phenotypes are often highly penetrant (e.g., Canli et al., 2005) and can be surprisingly large. This allows the investigation of substantially smaller sample sizes and makes it possible to detect significant genotype effects in the absence of overt behavioral differences (e.g., Roffman et al., 2008a). For these reasons, the study of genetic mediation using neuroimaging-based endophenotypes holds promise for uncovering susceptibility genes, mechanisms of illness, and targets for intervention (Hariri et al., 2006).

**Figure 5:**
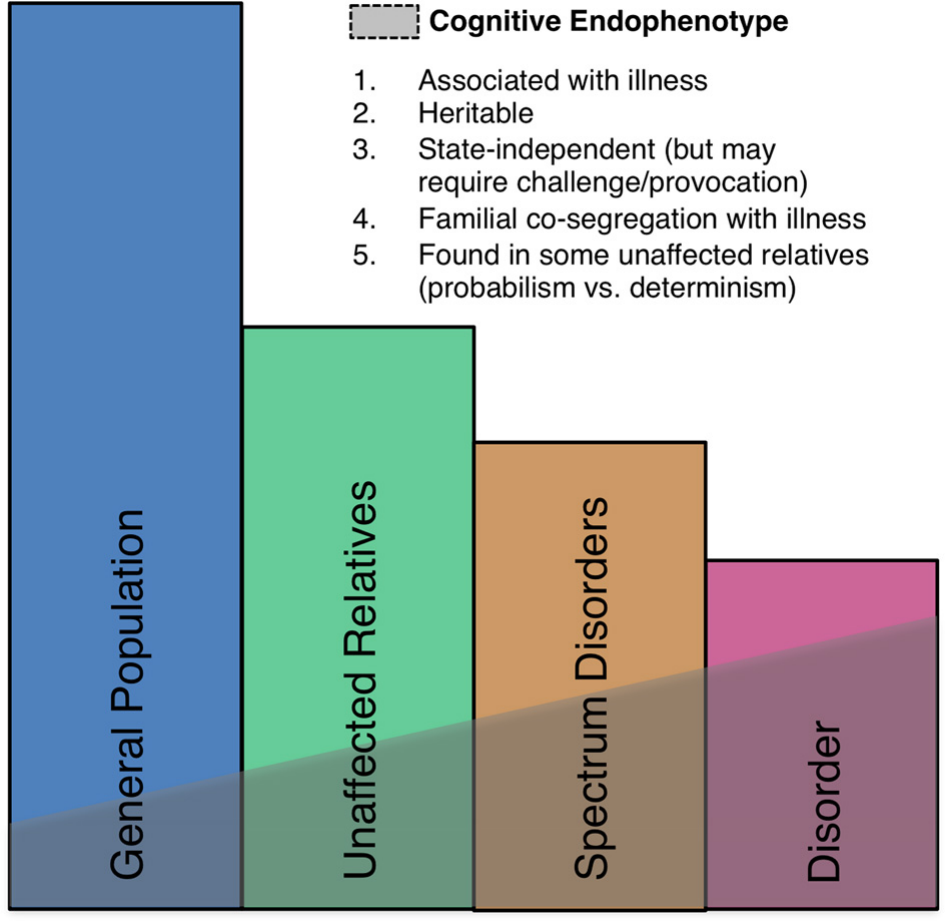
**A schematic illustration of the endophenotype concept.** Shaded areas indicate the presence of the endophenotype in affected patients, individuals with spectrum disorders, syndromally-unaffected family members and the general population. Criteria taken from Gould and Gottesman (2006).

Neural markers of errors, such as the ERN, meet several important criteria as endophenotypes (Gottesman and Gould, 2003) including high heritability based on both sibling (Albrecht et al., 2008) and twin (Anokhin et al., 2008) studies, established neuroanatomical and neurochemical substrates, and association with psychiatric disorders, though they are also seen in the general population (Figure 5). There is also growing evidence of genetic mediation of neural error markers both in health and psychopathology.

## Genetic variation influences error processing in health and neuropsychiatric disorder (see Table 1 for a summary).

### The role of dopamine in error processing

Empirical work and theory document a critical role for the dopaminergic system, particularly D2-like DA receptors, in reinforcement learning (Schultz et al., 1997). Reinforcement learning theory has been extended to encompass error-based reinforcement learning and, as described above, both the ERN and error-related dACC activation are seen to arise from this DA dependent mechanism (Holroyd and Coles, 2002). Converging lines of evidence support a role for DA in error processing. Individuals with Parkinson's Disease, which is caused by a loss of midbrain DA neurons, have a blunted ERN (Falkenstein et al., 2001; Ito and Kitagawa, 2006; Willemssen et al., 2009). Pharmacological manipulation of DA affects neural responses to errors. Haloperidol, a DA D2 receptor antagonist, blunted ERN amplitude in two studies (Zirnheld et al., 2004; De Bruijn et al., 2006), while D-amphetamine, an indirect DA agonist, increased it (De Bruijn et al., 2004). Additional support for a DA-dependent mechanism of error processing comes from findings that genetic polymorphisms affecting DA neurotransmission influence error markers in both health and neuropsychiatric disorders.

**Table 1:** **Genetic polymorphisms affecting EEG and fMRI error markers**.

**Polymorphism**	**Effect on error markers**
DRD2-TAQ-IA (rs1800497)	Reduced dACC activation in A1 allele carriers (Klein et al., 2007b), increased ERN in A1 carriers (Meyer et al., 2012), no effect on the ERN (Althaus et al., 2009).
DRD4 C-521T (rs1800955)	Increased ERN in T-allele carriers (Kramer et al., 2007).
DRD4 exon 3 VNTR	Reduced ERN in 7R allele carriers (Biehl et al., 2011).
DAT1 3′-UTR VNTR	Increased ERN (Meyer et al., 2012), increased Pe (Althaus et al., 2010) and decreased Pe (Biehl et al., 2011) in 9R allele carriers.
COMT Val^158^Met (rs4680)	In *Val* allele carriers increased ERN (Osinsky et al., 2012) or a trend to an increased ERN (Kramer et al., 2007), no effect on the ERN but increased Pe in *Met* homozygotes (Frank et al., 2007).
MTHFR 677C>T (rs1801133)	Reduced dACC activation in T-allele carriers (Roffman et al., 2011a,b).
Serotonin Transporter 5-HTTLPR	Increased ERN in short allele homozygotes (Fallgatter et al., 2004), no effect on the ERN (Olvet et al., 2010).
5-HT1A Receptor C-1019G (rs6295)	Reduced ERN in G-allele carriers (Beste et al., 2010).
BDNF Val^66^Met (rs6265)	Reduced ERN and post-error slowing in *Met* allele carriers (Beste et al., 2012).
NPSR Asn^107^Ile (rs324981)	Increased ERN and post-error slowing in Ile carriers (Beste et al., 2013).

### DRD2 TAQ-IA

The DA D2 receptor gene is a risk gene for schizophrenia (Shi et al., 2008) and the polymorphism, *TAQ-1A* (rs1800497), which is associated with schizophrenia (Parsons et al., 2007), predicts response to treatment with risperidone (Ikeda et al., 2008) and aripiprazole (Kwon et al., 2008). An fMRI study of healthy individuals (Klein et al., 2007b) found that *A1* allele carriers, with putatively reduced striatal DA receptor density (Pohjalainen et al., 1998; Jonsson et al., 1999; Ritchie and Noble, 2003), showed decreased dACC activation in response to errors and decreased avoidance learning, suggesting that they were less efficient in learning from errors. *A1* allele carriers also showed decreased functional connectivity of the dACC and striatum. With regard to the ERN, there are conflicting reports of no association with *DRD2 TAQ-IA* (Althaus et al., 2009) and an increased ERN amplitude in *A1* allele carriers (Meyer et al., 2012).

### DRD4 C-521T

The DA D4 receptor gene (*DRD4*) is also a candidate gene for schizophrenia (Shi et al., 2008) and the −521 single nucleotide polymorphism (SNP) refers to a *C-*to-*T* substitution in the *DRD4* promoter region (rs1800955) with the *T* allele resulting in 40% less transcriptional efficiency (Okuyama et al., 1999). The *DRD4 -521C* allele has been associated with schizophrenia (Okuyama et al., 1999; Xing et al., 2003; Allen et al., 2008) and healthy individuals homozygous for the *C* allele showed a decreased ERN and decreased post-error slowing compared to *T* homozygotes (Kramer et al., 2007).

### DRD4 EXON 3 VNTR

Another *DRD4* polymorphism linked to error processing consists of a variable number of tandem repeats of a 48-base-pair sequence in the third exon (Van Tol et al., 1992). The most frequently occurring numbers of repeats are 4 (*4R*; 70%), 7 (*7R*; 20%), and 2 (*2R*; 5%) (Asghari et al., 1995). The *7R* allele has been associated with higher risk of OCD (Taj et al., 2013), with tics in OCD (Cruz et al., 1997), and with reduced ERN amplitude, but comparable Pe in healthy individuals (Biehl et al., 2011).

### The DA transporter *(DAT1) 3'-UTR VNTR*

DAT1 plays a key role in regulating DA neurotransmission by facilitating re-uptake of DA in the synaptic cleft (Jaber et al., 1997). A polymorphism in the 3′-untranslated region (3′-UTR) of this gene consists of a variable number of tandem repeats of a 40-base-pair sequence, ranging from 3 to 11 copies of the repeated sequence, with the most common variants being 9 (*9R*; 24%) and 10 (*10R*; 70%) repeats (Vandenbergh et al., 1992). Carriers of the *9R* allele have increased levels of DAT1 in the striatum (Van Dyck et al., 2005; Van De Giessen et al., 2009) and a trend for increased risk of OCD based on meta-analysis (Liu et al., 2012). The *9R* allele has also been associated with a larger ERN (referred to as ΔERN in Meyer et al., 2012), and a larger Pe in one study (Althaus et al., 2010), but a smaller Pe in a second study (Biehl et al., 2011).

### COMT Val^158^Met

A G-to-A SNP in the catechol-O-methyltransferase (COMT) gene leads to a valine-to-methionine substitution (*COMTVal*^*158*^*Met*, *rs*4680). COMT metabolizes released DA and the *Met* allele significantly reduces COMT activity, leading to higher DA. The *COMTVal*^*158*^*Met* polymorphism has been studied extensively in relation to schizophrenia, and several meta-analysis have argued against association (Fan et al., 2005; Munafo et al., 2005; Okochi et al., 2009). While *COMT Val*^*158*^*Met* primarily affects DA availability in the prefrontal cortex (Egan et al., 2001; Craddock et al., 2006b), it may also have downstream effects on midbrain DA (Meyer-Lindenberg et al., 2005). Studies of error processing have yielded inconsistent findings, showing an increased amplitude of the ERN in *Val* allele carriers (Osinsky et al., 2012), only a trend-level enhancement of the ERN in *Val* compared to *Met* homozygotes (Kramer et al., 2007), and no effect of *COMT Val*^*158*^*Met* on the ERN but an increased Pe in *Met* homozygotes compared to *Val* carriers (Frank et al., 2007).

### MTHFR 677C>T

The hypofunctional *677T* variant in the methylenetetrahydrofolate reductase gene (*MTHFR 677C*>*T, rs*1801133) has been associated with increased risk for schizophrenia (Gilbody et al., 2007; Allen et al., 2008), executive dysfunction in schizophrenia (Roffman et al., 2008b), and negative symptoms (Roffman et al., 2008c). Several steps in the DA lifecycle rely on methylation reactions regulated by MTHFR (Friso et al., 2002) and each copy of the *T* allele reduces MTHFR activity by 35% (Frosst et al., 1995). The *T* allele has been shown to reduce dorsolateral prefrontal cortex fMRI activation during working memory performance in schizophrenia, both on its own, and via epistatic interactions with the low-DA *COMT 158Val* allele, supporting a role of *MTHFR* in prefrontal DA signaling (Roffman et al., 2008a). There is also indirect evidence linking *MTHFR* to striatal DA. MTHFR is a key enzyme in the metabolism of homocysteine, which has toxic effects on DA neurons in the striatum of rats (Imamura et al., 2007). In alcohol dependent individuals, *MTHFR 677T* has been associated with higher plasma levels of homocysteine and increased risk of withdrawal seizures, which were interpreted to reflect the neurotoxic effects of homocysteine on the mesencephalic DA system (Lutz et al., 2006, 2007; Lutz, 2008).

In a prior study of executive function in schizophrenia (Roffman et al., 2008b), *MTHFR 677T* was specifically related to a behavioral index of error processing, namely increased perseverative errors on the Wisconsin Card Sort Test, which reflect a failure to use feedback to adjust behavior. Recent work has demonstrated significant *677T* allele-related reductions in error related fMRI activation of the dACC in healthy individuals and in two independent samples of patients with schizophrenia (Roffman et al., 2011a,b). The reductions in dACC activation were linearly related to allele dose regardless of diagnosis (Roffman et al., 2011b). This suggests that *MTHFR 677T* mediates error processing in both health and schizophrenia.

### Other genetic variation related to error processing

#### The serotonin transporter gene (5-HTTLPR)

Evidence linking serotonin to ACC function and structure comes from studies of a functional length variation in the transcriptional control region of the serotonin transporter gene in healthy individuals. This polymorphism was associated with differences in the anatomy and function of the amygdala- rACC circuit in healthy individuals (Pezawas et al., 2005), which has been implicated in generating and learning from negative affect (for review see Drevets, 2000; Baxter and Murray, 2002; Zald, 2003). This learning may extend to errors since both the rACC and amygdala respond to errors, and together, activation in these structures predicts error rate (Polli et al., 2008, 2009).

More direct evidence of a role for this polymorphism in error processing are findings of a significantly increased ERN amplitude and a trend to increased Pe amplitude in short allele homozygotes, who presumably produce less serotonin transporter transcript, compared to long allele homozygotes (Fallgatter et al., 2004). A larger study, however, failed to replicate the association of *5-HTTLPR* genotype with ERN amplitude (Olvet et al., 2010).

#### 5-HT1A receptor gene C-1019G

A SNP present in about a third of the population consisting of an extra base pair in the promoter region of the *5-HT1A* receptor gene (*C-1019G*, rs6295) has been associated with reduced ERN and post-error slowing (Beste et al., 2010). The presence of a guanine nucleotide prevents binding of repressor proteins, which leads to enhanced gene expression and reduced serotonergic transmission (Lemonde et al., 2003). The *G* allele has been linked to increased risk of schizophrenia (Huang et al., 2004) and to worse treatment outcomes (Reynolds et al., 2006; Mossner et al., 2009), but a meta-analysis reported no association with schizophrenia (Kishi et al., 2011).

#### BDNF Val^66^Met

The brain-derived neurotrophic factor (BDNF) is a nerve growth factor thought to facilitate synaptic connections in the brain (Cohen-Cory et al., 1996). A SNP in the eponymous gene, which encodes for BDNF, results in valine-to-methionine substitution in the prodomain of the protein (*BDNF Val*^*66*^*Met*, rs6265) that leads to reduced activity-dependent secretion of BDNF (Egan et al., 2003). One meta-analysis found an elevated risk for schizophrenia in homozygous *Met* carriers (Gratacos et al., 2007), but another did not (Kanazawa et al., 2007). The *Met* allele has been associated with earlier onset of schizophrenia (Chao et al., 2008) and reductions of ERN amplitude and post-error slowing in healthy individuals (Beste et al., 2012).

#### NPSR Asn^107^Ile

Neuropeptide S (NPS) is a 20 amino-acid peptide that modulates stress and arousal (Okamura and Reinscheid, 2007). An A-to-T substitution at position 107 of the gene encoding for the NPS receptor (NPSR) leads to an amino acid exchange from Asn to Ile (Asn^107^Ile, rs324981) and increases the efficacy of NPS about tenfold (Reinscheid et al., 2005). The T allele is thought to be related to anxiety disorders, particularly panic disorder (Domschke et al., 2011), and is associated with an increased ERN and more pronounced post-error slowing in healthy individuals (Beste et al., 2013).

## Challenges to the study of neural indices error processing as endophenotypes

### Failures of replication in imaging-genetics studies

Failures of replication are extremely common in imaging-genetics studies and represent a major challenge. Imaging-genetics findings are often based on relatively small samples and negative results are much less likely to be published. Smaller samples are often justified based on evidence that neuroimaging-based endophenotypes result in increased effect sizes in studies of genetic variation than behavior or diagnosis. The pragmatic justification is that neuroimaging studies are costly and require considerable infrastructure to accomplish. Relatively small and comprehensive studies can identify the most promising cognitive constructs and endophenotypes, which can then be exported for use in larger multisite studies of patients, relatives, and racially and ethnically homogeneous groups as has been done for studies of other putative cognitive endophenotypes (e.g., Turetsky et al., 2008; Radant et al., 2010). Studies in developing countries can complement and extend these efforts by identifying overlapping and distinct genetic contributions in non-western populations (e.g., Chan et al., 2010). To protect against false positive associations in smaller studies, it is often advisable to investigate the effects of only a limited set of polymorphisms that are selected based on stringent criteria and to seek convergence in the data. This strategy can maximize scientific yield while minimizing the risk of spurious findings by focusing on a hypothesis-driven set of loci that affect specific neural mechanisms, and are most likely to affect a particular endophenotype or set of related endophenotypes given the current state of knowledge. A limitation to this approach is that it will not represent the full complement of genes that influence the phenotypes of interest.

### Methodological differences across studies may lead to conflicting findings

Another challenge in the error processing literature is that the definition and measurement of neural indices of error processing vary across studies. The ERN, for example, can be defined based on the peak of negativity in either the error waveform alone or in the difference (error vs. correct) waveform. It is arguable which method is more valid. Such measurement differences can affect study outcomes as can be illustrated in OCD, which is often characterized by exaggerated neural responses on both correct and error trials. Several studies reporting an increased ERN in OCD, or in non-clinical populations with OCD symptoms, defined it using only the error trial (Gehring et al., 2000; Johannes et al., 2001; Hajcak and Simons, 2002; Endrass et al., 2008, 2010). In at least two of these studies, the waveform for correct trials was also more negative in OCD participants than controls (referred to as the correct-related negativity or CRN). Consequently, had the ERN been defined as the difference waveform, it might not have been greater in OCD patients than controls.

Methodological differences may also contribute to discrepancies in fMRI results. For example, most standard fMRI analysis techniques assume a shape to the hemodynamic response. While this is a statistically powerful technique when the models are correct, a single assumed model is unlikely to be valid across all brain regions and stimulus types (Duann et al., 2002) and, importantly, model inaccuracies may lead to the misattribution of activity to adjacent events (Manoach et al., 2003). Thus, it is possible that in some studies, increased ACC activation on error trials may reflect greater activation while planning or preparing the response rather than an exaggerated response to the error. Finite Impulse Response (FIR, Burock and Dale, 2000; Jansma et al., 2013) or other models that make no *a priori* assumptions about the shape of the hemodynamic response, may more accurately distinguish preparatory activation from error-related activation, and can also be used to evaluate the temporal characteristics of the hemodynamic response, which may differ between study groups (e.g., Dyckman et al., 2011).

Conflicting findings of error processing deficits in particular disorders may also arise from a number of other factors such as the use of different tasks and levels of difficulty, the characteristics of the samples studied such as whether certain symptoms are present, treatment with medications, and task performance (e.g., Mathews et al., 2012). By affecting neurotransmitter systems that mediate error responses, medications such as antipsychotics, selective serotonin reuptake inhibitors, and antidepressants may affect outcome measures and obscure group differences and effects of genetic variation. Task performance is also important to consider in evaluating error indices. More frequent errors are also more predictable, and ERN amplitude is thought to code the degree to which errors are unexpected (Holroyd and Coles, 2002; Brown and Braver, 2005), consistent with findings of inverse correlations between error rate and ERN amplitude (Gehring et al., 1993; Hajcak et al., 2003; Agam et al., 2011). The same may be true for dACC activation, which also correlates with error rate in some studies (e.g., Polli et al., 2008). Thus, different error rates in patient and control samples or in pre- and post-treatment conditions (e.g., Sokhadze et al., 2012a) represents a potential confound that could be statistically controlled (e.g., Polli et al., 2008). For example, in several ERN studies ASD participants had a higher error rate than controls (e.g., Sokhadze et al., 2010, 2012b; South et al., 2010), making it unclear whether the blunted ERN reflected more frequent errors or deficient error recognition and signaling.

### Limitations to the clinical utility of error processing endophenotypes

Unlike neurodegenerative disorders such as Alzheimer's Disease, which are associated with specific neuropathologies, neuropsychiatric disorders likely have multiple overlapping etiologies and neuropathologies. Consequently, neuropsychiatric disorders lack sensitive and specific pathophysiological markers such as amyloid beta protein, which is specific to Alzheimer's Disease, is thought to cause the associated dementia, and can be measured *in vivo* to assess the risk of developing symptoms and response to therapy (Klunk, 2011). Error processing endophenotypes, in contrast to amyloid beta protein, do not index a known, specific neuropathology and their diagnostic specificity remains to be established. Also, unlike amyloid beta protein, whose presence is usually associated with pathology, neural error markers are normally present and abnormality is defined as statistical deviation of their parameters from the norm, which varies from study to study. Measurement variability, the lack of consensus definitions of error markers, and the absence of large-scale studies make it difficult to define clear cut-offs for “normality.” In addition, error processing endophenotypes, such as a blunted or exaggerated ERN or error-related dACC activation, are only probabilistically associated with illness, they do not determine illness. The cognitive dysfunction that they index may make illness more probable, but is likely just one of a number of cumulative hits of relatively small effect. Given that we lack a comprehensive understanding of the genetic contributions to these markers, it is difficult to distinguish “false positives” (i.e., abnormal error markers in the absence of genetic risk in an otherwise healthy individual) from valid genetic vulnerability for a disorder that has not manifested itself for environmental reasons or due to other, protective, epigenetic or genetic factors. Similarly, because endophenotypes are only probabilistically related to illness and current diagnostic categories are heterogeneous, they may only be present in a subset of individuals within a given diagnostic group.

### Establishing the clinical relevance of error processing deficits

While there is clear evidence that deficient error processing is associated with symptoms and functional outcome in neuropsychiatric disorders, further research is required to fully elaborate the bases of these relations both within and across disorders. If, as we and others have proposed, deficient error processing mediates the pathway between genetic predisposition and illness by interfering with adaptive responses to outcomes (e.g., Olvet and Hajcak, 2008), early intervention and prevention may be possible, for example, in individuals at high risk for schizophrenia who show a blunted ERN (e.g., Laurens et al., 2010; Perez et al., 2012; Simmonite et al., 2012). It may also be possible to intervene to prevent relapse. Two recent studies demonstrate that error-related dACC activation predicts relapse and time to relapse in cocaine-dependent individuals (Luo et al., 2013) and recidivism in criminal offenders (Aharoni et al., 2013). These findings provide a rationale for the development of interventions to ameliorate error processing deficits in neuropsychiatric disorders as well as other populations characterized by repetitive, maladaptive behaviors.

## Conclusions

The existing literature on error processing allows the generation of biologically plausible hypotheses concerning the effects of genetic variation on well-validated and heritable indices of error processing that are abnormal in neuropsychiatric disorders, show evidence of diagnostic specificity, contribute to disability, and are thought to be mediated by specific neural mechanisms. Understanding the genetic mediation and mechanisms of error processing deficits in neuropsychiatric disorders may eventually lead to the development of specifically targeted interventions and enable the use genetic information to identify individuals most likely to benefit from these treatments. This can substantially reduce outcome variability, thereby increasing power, and reducing the required sample size and cost of treatment trials. The findings of imaging-genetics investigations may also provide novel neural and behavioral targets for treatment and sensitive surrogate markers of treatment response. Treating error processing deficits may significantly affect functional outcome in neuropsychiatric disorders or possibly even prevent onset or relapse since error signals provide crucial information for flexible adaptation to changing environments, and deficits in learning from errors, as indexed by abnormal neural responses and reduced behavioral adaptation, likely substantially contribute to rigid, perseverative, and maladaptive patterns of behavior. Given the dearth of effective interventions for cognitive deficits in neuropsychiatric disorders, this represents a promising approach.

### Conflict of interest statement

The authors declare that the research was conducted in the absence of any commercial or financial relationships that could be construed as a potential conflict of interest.
